# The Influence of CPAP Therapy on Basal Metabolic Rate and Physical Activity in Obese Patients with Obstructive Sleep Apnea

**DOI:** 10.3390/nu15204446

**Published:** 2023-10-20

**Authors:** Dimitra Siopi, Paschalis Steiropoulos

**Affiliations:** 1 Department of Pulmonology, General Hospital “G. Papanikolaou”, 57010 Thessaloniki, Greece; 2 MSc Programme in Sleep Medicine, Medical School, Democritus University of Thrace, 68100 Alexandroupolis, Greece

**Keywords:** obstructive sleep apnea, obesity, basal metabolic rate, physical activity, CPAP treatment

## Abstract

Background: Energy balance in Obstructive Sleep Apnea (OSA), a disease closely related to obesity, is disturbed, and physical activity levels are impaired. The role of Continuous Positive Airway Pressure treatment (CPAP) in alleviating the disruptions mentioned above is questioned. The objective of this study is to explore changes in energy expenditure (EE) and physical activity (PA) in obese patients with OSA after CPAP treatment. Methods: An assessment of Basal Metabolic Rate (BMR) via indirect calorimetry (IC) was performed on 24 obese patients (male in the majority (87.5%), mean age of 52.4 ± 9.8 years), newly diagnosed with moderate–severe OSA by polysomnography, at 4-time points: at baseline, at CPAP titration, at the 1-month and the 3-month follow up. Physical activity levels were subjectively estimated using the International Questionnaire of Physical Activity (IPAQ) before and after 3 months of adherent CPAP application. Results: BMR significantly decreased after CPAP treatment (1926 ± 537.8 kcal/d at baseline, 1790 ± 493.7 kcal/d at CPAP initiation, 1680.3 ± 600.8 kcal/d at 1 month, and 1581.3 ± 478.9 kcal/d at 3 months follow up (*p* < 0.001)). No significant changes in IPAQ were observed over time: baseline median IPAQ: 3894 (1487.5–11,755.5) total MET·min·wk^−1^, 3-month median IPAQ: 3900 (1512–11,824.5) total MET·min·wk^−1^. Conclusions: CPAP has an appreciable time effect on the BMR of obese patients with moderate–severe OSA. However, this change is not accompanied by a significant increase in physical activity levels.

## 1. Introduction

Obesity is well recognized as a global epidemic associated with markedly increased morbidity and mortality. Over the last few decades, its influence over both developed and developing countries has posed a significant burden on healthcare systems [1,2].

OSA is the most common sleep-related respiratory disorder, characterized by repetitive episodes of upper airway collapse, leading either to a reduction or to complete cessation of airflow in the presence of breathing efforts. Those episodes are associated with recurrent oxyhemoglobin desaturation events, referred to as “intermittent hypoxia”, a major hallmark of OSA [3,4]. The importance of this common disorder in the general population is constantly highlighted due to its association with cardiovascular diseases, metabolic disorders, impaired neurocognitive function, and excessive daytime sleepiness [5]. 

An indisputable relationship exists between OSA and obesity, the latter being considered a leading OSA risk factor. This relationship, thus, seems to be bidirectional and complex. There are many mechanisms by which excess weight may predispose to OSA. Some of them are well established, such as upper airway fat deposition, abdominal fat presence and altered respiratory mechanics, leptin resistance, and breathing instability [6,7]. The other direction of the relationship (OSA favoring a positive energy balance) seems less clear, with a rising hypothesis involving decreased physical activity and neurohormonal mechanisms that control hunger [7,8].

BMR represents the energy required to maintain basic functions and metabolic activity and is the largest component of overall EE. OSA seems to result in an increased BMR, independent of weight [8,9,10], but findings are inconsistent concerning the impact of sleep-disordered breathing on Sleeping Metabolic Rate (SMR) [8,11] and 24 h EE [11]. Different methods (canopy hood, face mask, whole room IC) have been used to examine all the related parameters (BMR, Sleeping EE, 24 h EE, and the BMR/sleeping EE ratio) with contrasting results. A reported OSA-related rise in EE might be attributed to increased sympathetic activity and breathing efforts as indicated by associated plasma and urine markers elevation [8]. At the same time, OSA may favor an increase in energy uptake due to behavioral or neurohormonal changes [12]. There is a history of weight gain over the year prior to OSA diagnosis [13], and when lifestyle interventions are followed, a smaller decrease in Body Mass Index (BMI) and fat mass is achieved in OSA patients than in non-OSA BMI-matched individuals [14].

Treating OSA with CPAP would be expected to have a favorable impact on weight regulation. Surprisingly, some recent studies and a meta-analysis have proven weight gain following CPAP treatment with unclear underlying mechanisms [15,16]. It has been reported that the additional weight represents fat-free mass, which is considered a positive metabolic outcome [17]. Overall, there is still conflicting evidence concerning the impact of CPAP treatment or surgical therapies on EE and its clinical implications in OSA patients.

The change in PA following CPAP treatment is another field of argument, with related studies yielding conflicting results. Using different methods, some studies have reported no change in PA [18,19,20], whereas others have shown a progressive improvement [21,22].

The scope of the present study is to assess energy metabolism by estimating BMR, as well as self-recorded physical activity levels in obese OSA patients before and after treatment intervention with CPAP.

## 2. Materials and Methods

Participants: Patients were recruited from the Sleep Laboratory of the N.H.S. Department of Pulmonology, General Hospital “G. Papanikolaou”, Thessaloniki, Greece, from May 2020 until April 2022. They were all adult, obese patients (with a Body Mass Index (BMI) ≥ 30 kg/m^2^) referred with high suspicion of OSA, which was confirmed by in-laboratory polysomnography. Exclusion criteria included abnormal thyroid function, neuromuscular disease, Chronic Obstructive Pulmonary Disease (COPD), and the diagnosis of predominant central sleep apnea. This study was approved by the Institutional Review Board (Approval code: 1056/18.09.2019, Approval date: 8 September 2019). All patients provided written consent for participating.

Study Protocol: Anthropometric parameters (weight, height, BMI, neck, waist, and hip circumference), medical history, Epworth Sleepiness Scale (ESS) and self-assessed physical activity using the IPAQ were recorded at baseline. Arterial and venous blood samples were collected to be tested respectively for blood gases and lipidemic profile, uric acid, liver function, and C-reactive protein (CRP). Spirometry was used to assess pulmonary function. After in-laboratory polysomnography was performed, BMR was estimated via face mask IC.

In patients diagnosed with moderate–severe OSA (Apnea Hypopnea Index (AHI) ≥ 15 events/h) indicated for CPAP treatment, energy metabolism and physical activity were reassessed as follows: IC, in order to estimate BMR, was repeated after the first night of CPAP titration, after 1 month, and after 3 months of CPAP treatment.

Physical activity was re-evaluated by the IPAQ after 3 months of CPAP treatment.

CPAP adequate adherence was based on usage data from the machines’ memory cards. Only compliant patients (using CPAP > 4 h per night, at least 5 days per week), according to machine-derived data, were included in the analysis.

No dietary or exercise changes were advised during the study.

### 2.1. Polysomnography

Each included patient underwent in-laboratory, supervised diagnostic polysomnography using standard procedures. The polysomnography recordings were performed and staged according to AASM criteria [23]. The equipment used was the EMBLA digital system (EMBLA S7000, Embla Systems Inc., Broomfield, CO, USA). It included electroencephalogram, electrooculogram, chin and anterior tibialis electromyogram, electrocardiogram, nasal pressure transducer and oronasal thermistor, snoring detection by tracheal microphone, thoracic and abdominal respiratory effort recording.

Following diagnostic polysomnography, patients with moderate–severe OSA underwent in-laboratory CPAP titration, applied by a sleep technologist, according to AASM guidelines [24,25].

### 2.2. Basal Metabolic Rate

The Basal Metabolic Rate via indirect calorimetry was assessed 4 times as follows: (1) at the time of the diagnostic polysomnography (baseline), (2) after the first night of in-laboratory CPAP titration, (3) after 1 month, and (4) after 3 months of CPAP home treatment.

Subjects were asked to refrain from exercise and avoid alcohol and caffeine 48 h before the test. After a 12 h overnight fast, participants reported to the laboratory between 07:00 and 09:30 a.m. All patients were asked to rest in the supine position for 30 min at an ambient temperature of 23–26 °C [26].

Oxygen uptake (VO_2_) and carbon dioxide production (VCO_2_) were recorded breath by breath for 20 min after an initial 10 min habituation period using a face mask. Calibration, according to the manufacturer’s instructions, was completed before each test.

The equipment used was COSMED-Quark PFT, COSMED, Rome, Italy.

### 2.3. Physical Activity

Physical activity was self-assessed using the translated short version; there was seven-day recall period of the IPAQ. IPAQ is a valuable instrument validated in the adult Greek population, providing information on weekly time spent on many domains of physical activity (light, moderate, and vigorous intensity) [27,28]. Both continuous and categoric measurements of IPAQ were assessed at the time of the diagnosis (baseline) and after 3 months of CPAP treatment. The continuous indicator was expressed as the metabolic equivalent of the task total (MET) minutes per week. PA status was classified into 3 categories: low PA class with a total PA score < 600 MET·min·wk^−1^, moderate PA class with a total PA score ≤ 600 > 3000 MET·min·wk^−1^, and high PA class with a total PA score ≥ 3000 MET·min·wk^−1^.

### 2.4. Spirometry

Spirometry was performed according to the ERS-ATS guidelines [29] using COSMED Q-box.

### 2.5. Blood Samples

Venous blood samples were collected between 06:30 and 07:00 a.m. at baseline. Arterial blood samples were collected at rest, during wakefulness after the diagnostic polysomnography, with the examined patients sitting upright.

Statistical Analysis

Quantitative variables were presented as mean (SD) or median (IQR) values. Qualitative variables were expressed as absolute and relative frequencies (N, %). The normal distribution of the data was tested using the Kolmogorov–Smirnov and Shapiro–Wilk tests. The Spearman correlation coefficient was used to estimate the linear correlation of parameters with BMR and IPAQ at baseline. Comparison of BMR between the four time points was analyzed using repeated-measures analysis of variance (ANOVA), with time as a within-subject factor and the ESS, AHI, Rapid Eye Movement (REM) index, mean saturation, and minimum saturation measurements as between-subjects factors. Partial eta-squared (η^2^) was used as a measure of effect size. A repeated-measures analysis of variance (ANOVA) was performed to assess the effects of time on IPAQ and ESS, AHI, REM index, mean, and minimum saturation as between-subject factors. All statistical analyses were performed by IBM SPSS Statistics version 25.0. Values of *p* ≤ 0.05 were considered statistically significant.

## 3. Results

From 28 examined individuals who fulfilled the inclusion criteria, a total of 24 patients participated in the study. One patient was excluded because of poor compliance with CPAP treatment, and the other three were diagnosed with mild OSA not indicated for CPAP treatment. The clinical characteristics of the study participants are shown in Table 1. The majority of the sample was male (87.5%) and had a mean age of 52.4 ± 9.8 years. The mean baseline BMI (BMI_0_) was 40.9 ± 8.4 kg/m^2^. In addition, 41.7% of participants had arterial hypertension, 8.3% had diabetes mellitus, 8.3% had coronary artery disease, 20.8% had chronic atrial fibrillation, 20.8% had dyslipidemia, and 16.7% had other comorbidities. The mean ESS was 9.9 ± 5.5, the mean AHI was 70.6 ± 30.8 events/h, and the mean REM index was 40.4 ± 29.1 events/h. Mean and minimum oxygen saturation values during sleep were 88.8 ± 3.7% and 69.3 ± 11, respectively. Time spent in sleep with SpO_2_ < 90% was 40.9± 30.5%. The mean BMI at 3 months (BMI_3_) was 40.91 ± 8.38 kg/m^2^.

No statistically significant correlations were found between BMR measurement at baseline and ESS or sleep parameters: AHI, REM index, mean, and minimum oxygen saturation, time < 90%. Similar results were also found for IPAQ measurement at baseline, as no significant correlation was found between IPAQ and ESS, AHI, REM index, mean and minimum saturation, and time < 90% (Table 2).

**Figure 1:**
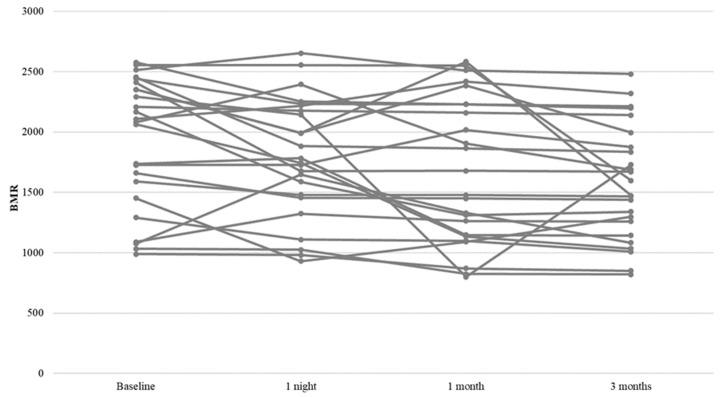
Profile plot for BMR change.

To evaluate differences in BMR over time, ANOVAs for repeated measures were conducted. The within-subject factor was time, and the between-subject factors were ESS, AHI, REM index, mean and minimum saturation. The results showed a significant main effect of “time” on BMR [F_(3, 69)_ = 7.835, *p* < 0.001, η^2^ = 0.254], indicating a significant reduction of BMR over time. Specifically, LSD post hoc comparisons revealed a significant reduction in BMR after 1 night (*p* = 0.041), after 1 month (*p* = 0.012), and after 3 months (*p* < 0.001) compared to baseline values (Table 3 and Table 4). In addition, a significant reduction in BMR was also observed at 3 months compared to that at 1 night. No significant interaction was observed between time and ESS [F_(3, 66)_ = 0.674, *p* = 0.571, η^2^ = 0.030], AHI [F_(3, 66)_ = 0.018, *p* = 0.997, η^2^ = 0.001], REM index [F_(3, 66)_ = 0.936, *p* = 0.428, η^2^ = 0.041], mean [F_(3, 66)_ = 2.607, *p* = 0.059, η^2^ = 0.106], minimum saturation [F_(3, 66)_ = 1.194, *p* = 0.319, η^2^ = 0.051], and BMI [F_(3, 66)_ = 0.270, *p* = 0.847, η^2^ = 0.012] (Table 5) (Figure 1).

For the evaluation of weight differences over time, a repeated measures ANOVA was performed. The results did not show a significant change in participants’ weight from baseline measurement to the measurement after three months [F_(1, 23)_ = 3.958, *p* = 0.059, η^2^ = 0.147] (Table 6 and Table 7).

**Figure 2:**
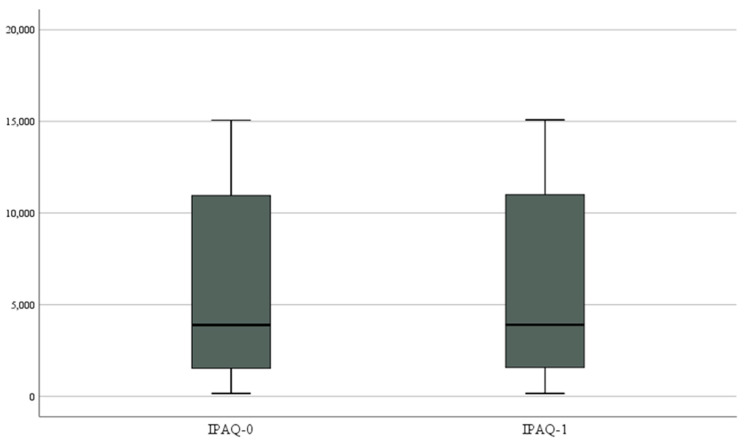
Box plot for IPAQ change.

The comparison (ANOVAs) over time in terms of physical activity is shown in Table 8 and Table 9. A non-significant increase in physical activity was found [F_(1, 23)_ = 3.792, *p* = 0.064, η^2^ = 0.142]. No significant interaction was found between time and ESS [F_(1, 22)_ = 0.203, *p* = 0.657, η^2^ = 0.009], AHI [F_(1, 22)_ = 0.059, *p* = 0.810, η^2^ = 0.003], REM index [F_(1, 22)_ = 0.493, *p* = 0.490, η^2^ = 0.022], mean [F_(1, 22)_ = 0.062, *p* = 0.805, η^2^ = 0.003], minimum saturation [F_(1, 22)_ = 0.023, *p* = 0.882, η^2^ = 0.001], and BMI [F_(1, 22)_ = 0.392, *p* = 0.538, η^2^ = 0.017] (Figure 2).

## 4. Discussion

The relationship between obesity, sleep-disordered breathing, and energy balance is complex and not well-studied. Data on EE of OSA patients and the impact of CPAP or other forms of therapy are scarce and conflicting.

Resting EE examined by different measuring methods and protocols is reported higher in OSA patients than in matched controls in many studies [8,10,11,30], whereas other researchers failed to prove such a connection [9,31]. Canopy hood, face mask, or whole room I.C. have been used to estimate BMR, SMR, 24 h EE, and sleep EE/BMR ratio.

In four out of six cross-sectional studies, BMR measured with IC was higher in OSA patients than in controls, even after adjustment for BMI and age [8,9,10] or just age [32]. Arousals, as well as the increased breathing effort against an occluded upper airway, may have an energy cost. Elevated sympathetic activity, as reflected by related plasma and urine markers, could explain augmented sleeping EE in OSA patients, even in a moderate degree of sleep-disordered breathing [8]. Other concepts, such as the adaptive decrease of thermogenesis, have not been thoroughly tested in OSA. The role of sleep deprivation seems important since it can lead to endocrine and immunological dysregulation, elevated cortisol and catecholamine levels, and influence hunger and appetite. Whether the same mechanisms are implicated in OSA is unknown [31,33].

Findings on SMR and 24 h EE are inconsistent [8,11,30,34]. Sleeping EE in OSA patients has been found to be higher than wakefulness EE, with the role of increased sympathetic activity highlighted [11]. In a study assessing 24 h EE using a chamber for IC, 24 h EE was higher in patients with severe OSA compared to individuals displaying a less severe degree of sleep-related breathing disturbance. Sleep EE also tended to be higher in OSA patients compared to simple snorers [8]. Lin et al. found the mean sleep EE, the ratio of mean sleep/BMR, as well as the lowest sleep EE/BMR ratio to be significantly higher in moderately severe or severe OSAS patients than in healthy control subjects [30].

Higher BMR values in OSA patients have been correlated with AHI/RDI and nocturnal hypoxemia [35,36]. A negative relationship between nocturnal desaturation and differences in measured 24 h EE and SMR has also been reported [33]. Such a correlation was not found in our study, most likely because of the small number of enrolled patients.

An important finding of our study is the effect of CPAP treatment on BMR, resulting in a reduction, both short-term (one night effect) and long-term (after 1 month and 3 months), with a rising time effect. This is in accordance with many studies that reported reduced EE following CPAP implementation. In their study, apart from investigating sleeping EE in OSA patients, Stenölf et al. showed a decrease in sleep EE and EE variability during sleep after three months of CPAP treatment. However, Resting Metabolic Rate (RMR) and 24 h EE did not change [8]. A significant BMR reduction of 5% has been reported by Tachikawa et al. in a comprehensive assessment after 3 months of CPAP treatment, demonstrating a possible causal effect, as the researchers associated the decrease in BMR with the decrease in urine norepinephrine, apart from CPAP adherence. The role of increased sympathetic activity ruled out by therapy could mediate a high BMR in OSA, according to the researchers [15]. Other therapeutic interventions, such as treatment by laser-assisted uvuloplasty, have also been reported to induce a subsequent fall of sleep EE [33].

Studies that did not demonstrate any change at all may have been underpowered [16,37]. A pilot cross-over placebo-controlled trial of active 2-month CPAP vs. sham CPAP treatment showed an increase in SEE and 24 h EE. The researchers suggested that CPAP might counterpoint the decreased adaptive thermogenesis in OSA, but the study size was relatively small (three patients), and there was no randomization [38].

There is emerging evidence that CPAP treatment results in a rise of 0.5–1 kg following treatment. The implication that BMR reduction after CPAP treatment favors a positive energy balance has been postulated [39,40]. According to recent evidence, this weight change reflects fat-free mass, making the response to CPAP a favorable metabolic outcome [17]. In our study, no significant weight changes after 3 months of treatment were detected, but we did not conduct a body composition assessment. The significant BMR changes recorded in our study population could not be attributed to weight changes.

In another study, BMR was similarly reduced after CPAP implementation in both weight gainers and non-weight gainers [15].

Another objective of our study was to record the level of PA before and after 3 months of treatment. Many studies using either objective methods or questionnaires have negatively related OSA and physical activity after controlling for age, sex, and ESS [30,41,42]. As stated, the high prevalence of obesity among OSA patients might be a confounding factor. Obesity per se could play an important role in influencing PA levels in OSA patients [43,44]. In our study, PA subjectively assessed by a questionnaire was not correlated with the level of sleepiness or OSA severity, not even AHI in REM sleep as previously reported [45].

Physical activity questionnaires are commonly used for practical reasons. The short-term IPAQ version is considered a reliable tool examined in the Greek population [26,27].

According to our study, self-reported physical activity of obese OSA patients does not change substantially after 3 months of compliant CPAP treatment despite the changes in BMR. This has also been reported in previous studies, using questionnaires as the case here [19] or objective methods such as actigraphy [18,46].

In a 6 months Randomized Control Trial of CPAP vs. sham CPAP on neurocognitive outcomes (APPLES), CPAP did not change physical activity, independent of confounders such as ESS, AHI, age, BMI, and CPAP adherence [19]. On the contrary, in a study using both actigraphy and questionnaires, there was a favorable result on PA after 1 month of CPAP compared to sham CPAP in OSA patients with type II diabetes [21]. Another prospective, longitudinal study reported an augmented PA using both IPAQ for perceived PA as well as pedometer steps per day for actual PA at three time intervals pre- and post-CPAP (3 and 8 months). The reported improvement was in parallel with an increment improvement in sleep quality [22].

Excessive sleepiness, one of the major clinical features of OSA, leads to decreased physical activity [22,41] but positively corresponds to CPAP treatment. The anticipation of such a positive effect on physical activity still needs to be met.

Our study has several strengths. Sleep-disordered breathing in all included patients was examined via polysomnography, allowing an accurate estimation of sleep disturbance. BMR was examined in three different time frames after CPAP treatment, showing a gradual improvement in adherent OSA obese patients. The simultaneous assessment of PA that did not change significantly despite the significant reduction in BMR is an important element in questioning the clinical implications of BMR changes. It also highlights the need for systematic lifestyle interventions and weight loss targeting strategies in order to encourage optimal outcomes.

There are several limitations in the current study. The most important ones are the low number of participants, the lack of a control group of patients not treated with CPAP, and the low representation of women observed in most studies, leaving questions concerning sex-based effects. The 3-month observation period could also be considered as not sufficient enough to lead to safe conclusions. Physical activity was not objectively assessed. Whereas objective monitoring would be preferable, several questionnaires on PA levels are widely used with good reliability and validity. One could also argue that seasonal changes may have influenced PA, a parameter not implemented.

## 5. Conclusions

In summary, the findings of our study indicate a statistically significant and time-related reduction in BMR in OSA-obese patients after adequate compliance with CPAP treatment. At the same time, only a minimal effect of OSA treatment on self-assessed PA was recorded. CPAP treatment alone does not seem sufficient to change impaired physical activity, and additional interventions are mandatory to encourage optimal outcomes. Enrolling obese OSA patients in well-organized programs and lifestyle modifications could lead to better results.

## Figures and Tables

**Table 1:** Patients’ characteristics at baseline.

Variables	Total (N = 24)
Gender Male, Ν (%)	21 (87.5)
Age (year)	52.4 ± 9.8
BMI_0_ (kg/m^2^)	40.9 ± 8.4
Hypertension, Ν (%)	10 (41.7)
Diabetes mellitus, Ν (%)	2 (8.3)
Coronary artery disease, Ν (%)	2 (8.3)
Chronic atrial fibrillation, Ν (%)	5 (20.8)
Dyslipidemia, Ν (%)	5 (20.8)
Other, Ν (%)	4 (16.7)
Epworth Sleepiness Scale (ESS)	9.9 ± 5.5
Apnea Hypopnea Index (AHI) (events/h)	70.6 ± 30.8
REM Index (events/h)	40.4 ± 29.1
Mean saturation (%)	88.8 ± 3.7
Minimum saturation (%)	69.3 ± 11
Time in sleep SpO_2_ < 90% (%)	40.9 ± 30.5
pH	7.4 ± 0.1
PO_2_ (mmHg)	76 ± 12.1
PCO_2_ (mmHg)	38.4 ± 5
Sat%	95.3 ± 3.1
HCO_3_ (mmol/lt)	24.2 ± 2.4
FEV_1_ (L)	3.1 ± 0.8
FVC (L)	3.9 ± 1
CHOL (mg/dL)	193.3 ± 30.6
LDL (mg/dL)	117.2 ± 29.8
HDL (mg/dL)	42.6 ± 11.9
TRIG (mg/dL)	189.6 ± 90.3
LDH (U/L)	209.8 ± 41.3
SGOT (U/L)	26.2 ± 8.9
SGPT (U/L)	35.5 ± 14.7
CRP (mg/L)	0.4 ± 0.1
Uric acid (mg/L)	6.7 ± 1.

FEV_1_: forced expiratory volume in the first second, FVC: forced vital capacity, CHOL: cholesterol, LDL: low-density lipoprotein, HDL: high-density lipoprotein, TRIG: triglycerides, LDH: lactate dehydrogenase, CRP: C-reactive protein, SGOT: serum glutamic oxaloacetic transaminase, SGPT: serum glutamate pyruvate transaminase, ALP: alkaline phosphatase.

**Table 2:** Spearman correlation coefficients for BMR and IPAQ measurements at baseline with sleep parameters.

	BMR (Baseline)	IPAQ (Baseline)
Epworth Sleepiness Scale	0.131 (0.541)	0.341 (0.103)
Apnea Hypopnea Index (AHI)	−0.138 (0.519)	0.398 (0.054)
REM Index	−0.114 (0.597)	0.259 (0.221)
Mean saturation	−0.229 (0.282)	−0.396 (0.055)
Minimum saturation	−0.279 (0.187)	0.015 (0.944)
Time < 90%	0.197 (0.357)	0.343 (0.102)

Spearman correlation coefficients are presented, with the corresponding *p*-values enclosed in parentheses.

**Table 3:** Mean (SD) values of BMR across time.

	Baseline	1-Night	1-Month	3-Months
BMR	1926 ± 537.8	1790.1 ± 493.7	1680.3 ± 600.8	1581.3 ± 478.9

Data are presented as mean ± SD.

**Table 4:** Analysis of variance of BMR repeated measures.

Source	Type III Sum of Squares	Mean Square	F	*p*	η^2^
Time	1,578,804.9	526,268.3	7.835	<0.001	0.254

**Table 5:** Analysis of variance with repeated measures of BMR with between-subject factors.

Source	Type III Sum of Squares	Mean Square	F	*p*	η^2^
Time × ESS	137,746.62	45,915.54	0.674	0.571	0.030
Time × AHI	3893.68	1297.89	0.018	0.997	0.001
Time × REM index	189,160.72	63,053.57	0.936	0.428	0.041
Time × Mean saturation	491,075.45	163,691.82	2.607	0.059	0.106
Time × Minimum saturation	238,570.27	79,523.42	1.194	0.319	0.051
Time × ΒΜΙ	56,104.95	18,701.65	0.270	0.847	0.012

**Table 6:** Mean (SD) values of weight across time.

	Baseline	1-Month	3-Months
Weight	119.1 ± 20.2	118.9 ± 20.2	118.7 ± 20.0

Data are presented as mean ± SD.

**Table 7:** Analysis of variance of weight repeated measures.

Source	Type III Sum of Squares	Mean Square	F	*p*	η^2^
Time	1.03	1.03	3.958	0.059	0.147

**Table 8:** Mean (SD) values of IPAQ across time. Changes in IPAQ across time.

	Baseline	3 Months
IPAQ	3894 (1487.5–11,755.5)	3900 (1512–11,824.5)

Data are presented as median (IQR).

**Table 9:** Analysis of variance of IPAQ repeated measures.

Source	Type III Sum of Squares	Mean Square	F	*p*	η^2^
Time × ESS	0.00	0.00	0.203	0.657	0.009
Time × AHI	51.13	51.13	0.059	0.810	0.003
Time × REM index	0.00	0.00	0.493	0.490	0.022
Time × Mean saturation	53.81	53.81	0.062	0.805	0.003
Time × Minimum saturation	0.00	0.00	0.023	0.882	0.001
Time × ΒΜΙ	0.00	0.00	0.392	0.538	0.017

## Data Availability

The data presented in this study are available on request from the corresponding author.
